# Dose inhibin B or anti-Müllerian hormone relate to precocious puberty in girls? result of a systematic review and meta-analysis

**DOI:** 10.1186/s13048-023-01302-2

**Published:** 2023-11-23

**Authors:** Mei Jiang, Ying Gao, Tiange Qu, Yuechen Ji, Yiwen Niu, Jiaxin Zhang, Ling Huang

**Affiliations:** 1 Beijing Research Institute of Chinese Medicine, Beijing University of Chinese Medicine, Beijing, China; 2 Department of Acupuncture, Wangjing Hospital of China Academy of Chinese Medical Sciences, Beijing, China; 3 Department of Dermatology, Dongzhimen Hospital of Beijing University of Chinese Medicine, Beijing, China; 4 School of traditional Chinese Medicine, Beijing University of Chinese Medicine, No.11 Beisanhuandong Road, Chaoyang, Beijing, 100029 P. R. China; 5 Department of Gastroenterology, Dongzhimen Hospital of Beijing University of Chinese Medicine, Beijing, China

**Keywords:** Precocious puberty, Inhibin B, anti-müllerian hormone, Premature thelarche, Premature pubarche, Premature adrenarche

## Abstract

**Backgrounds:**

Existing studies have investigated the relationship between the levels of serum inhibin B (INHB), anti-müllerian hormone (AMH) and precocious puberty in girls, but the results are inconsistent.

**Objective:**

The aim of this meta-analysis was to assess whether the INHB and AMH levels changed in girls with precocious puberty relative to healthy controls.

**Methods:**

PubMed, Embase, Cochrane Library and Web of Science were searched through June 2022. We included observational clinical studies reporting the serum levels INHB and AMH in girls with precocious puberty. Conference articles and observational study abstracts were included if they contained enough information regarding study design and outcome data. Case series and reports were excluded. An overall standard mean difference (SMD) between precocious puberty and healthy controls was estimated using a DerSimonian-Laird random-effects model.

**Results:**

A total of 11 studies featuring 552 girls with precocious puberty and 405 healthy girls were selected for analysis. The meta-analysis showed that the INHB level of precocious puberty [including central precocious puberty (CPP) and premature the larche (PT)] were significantly increased. While there was no significant association between precocious puberty [including CPP, PT, premature pubarche (PP) and premature adrenarche (PA)] and the level of serum AMH.

**Conclusion:**

Scientific evidence suggested that the INHB level, but not the AMH level, altered in girls with precocious puberty compared with healthy controls. Through our results we think that INHB level might be a marker for the auxiliary diagnosis of precocious puberty (especially CPP and PT). Therefore, it is important to evaluate and thoroughly investigate the clinical indicators (e.g., INHB) in order to ensure early diagnosis and medical intervention, and the risk of physical, psychological and social disorders in immature girls with precocious puberty is minimized.

## Introduction

Precocious puberty is defined as the development of secondary sexual characteristics in girls by the age of 8 and in boys by the age of 9 [1]. The incidence of precocious puberty in girls is increasing every year [2, 3]. Depending on whether the hypothalamic-pituitary-gonadal axis (HPGA) occurs or not, precocious puberty is classified as central precocious puberty (CPP) with the HPGA occurring driven by early increased gonadotropin-releasing hormone (GnRH) secretion, which accounts for roughly 80% [4], and peripheral precocious puberty (PPP) which is independent of GnRH secretion. In addition, incomplete precocity is the variation of precocious puberty, including premature thelarche (PT), premature pubarche (PP), premature menarche and premature adrenarche (PA) [5]. PT is characterized by isolated breast development without any other signs of sexual maturation, and it is caused by transient partial activation of the HPGA with excessive secretion of follicle-stimulating hormone (FSH) [6]. PA refers to an increase of adrenal androgen level independent of the HPGA, and it is identified as pubarche including the presence of pubic and axillary hair, apocrine body odor and acne before age of 8 years in girls and age of 9 years in boys [7].

In infancy and childhood girls, only a small number of follicles develops from the primordial to the antral stage, then to be atretic [8], while the number of growing follicles increases after 10 years [9]. Inhibin B (INHB) and anti-müllerian hormone (AMH) are dimeric glycoproteins belonging to the transforming growth factor-β (TGF-β) superfamily [10]. INHB is produced by the granulosa cells of developing ovarian follicles in response to gonadotropin stimulation,it peaks during the follicular phase of the menstrual cycle and reduces or is undetectable during the luteal phase [11–13]. As a marker of follicular development, serum level of INHB is low or unmeasurable in prepubertal girls and rises during puberty, increases sharply during Tanner stages I-III and is above adult level in Tanner stage III [14]. Thereafter, INHB level decreases during Tanner stages IV and V [14]. AMH, also known as müllerian-inhibiting substance, is also produced by granulosa cells of ovaries in women, rises in infancy and remains stable in early adulthood [15, 16]. However, according to the studies, serum level of AMH increases by 17% during 3 years prior to the pubertal onset. After the first 2 years of pubertal onset, AMH level decreases 30% [16]. These findings suggest that serum levels of AMH and INHB are essentially parallel to the HPGA. On this basis, we hypothesize that INHB and AMH might be utilized to distinguish precocious puberty from early pubertal stages. While the possible differences of INHB and AMH in terms of reproductive development and disorders, including precocious puberty, have not been properly evaluated.

Although examination of the ovarian maturation process is limited, early ovarian maturation may be a possible outcome in girls with precocious puberty. Even though it is difficult to pinpoint the precise mechanism of ovarian maturation, AMH and INHB are indicators of ovarian function, and may be used to detect girls at risk of precocious puberty. Therefore, serum levels of INHB and AMH may provide important information to diagnose precocious puberty. Knowledge of the potential associations between INHB, AMH and precocious puberty has public health significance for the diagnosis of precocious puberty in girls. As a result, this systematic review and meta-analysis examined the levels of INHB and AMH in girls with precocious puberty and healthy controls.

## Methods

### Reporting guidelines

This systematic review and meta-analysis was based and reported according to the Preferred Reporting Items for Systematic Reviews and Meta-Analyses (PRISMA) statement [17], registered with the PROSPERO database (http://www.crd.york.ac.uk/PROSPERO) under registration number: CRD42022345092. The study followed the guidelines of the Meta-analysis of Observational Studies in Epidemiology (MOOSE) [18].

### Search Strategy and information sources

PICO (population/intervention/comparison/outcome) included the following components: P (girls under 10), I (girls with precocious puberty), C (healthy girls), O (serum levels of INHB and AMH).In order to identify eligible studies, PubMed, Embase, Cochrane Library, and Web of Science databases were searched extensively through June 2022 (with English as the language; without location or journal restrictions)to identify published studies containing the following keywords: “precocious puberty” OR “sexual precocity” OR “premature puberty” OR “precocious sexual maturation” OR “early puberty” OR “earlier puberty” OR “early pubertal timing” OR “early maturation” OR “isolated premature thelarche” OR “premature thelarche” OR “premature pubarche” OR “premature adrenarche” OR “premature menarche” OR “early age of menarche” OR “PP” OR “CPP” OR “PPP” OR “IPT” OR “PT”AND“Anti-Müllerian hormone” OR “AMH” OR “Müllerian inhibiting substance” OR “MIS” OR “Inhibins” OR “Inhibin-beta subunits” OR “Inhibin B” OR “Inhibin-beta” OR “Inhibin β” OR “INHB”.

### Inclusion and exclusion criteria

The inclusion criteria for eligible studies were as follows:(1) original observational study of humans; (2) all patients involved in studies were diagnosed with precocious puberty before 8 years old; (3) all subjects involved in studies were younger than 10 years old; (4) studies focusing on the association between the levels of serum INHB, AMH and precocious puberty; and (5) with data on serum INHB or AMH levels of precocious puberty patients and healthy prepubertal girls of similar ages.

Below were the exclusion criteria: (1) non-clinical or animals studies; (2) reviews types of articles or case reports; (3) repeated publications; (4) subjects involved in studies were diagnosed with sex hormone releasing tumors, McCune Albright syndrome and Cushing’s Syndrome; (5) subjects involved in studies were diagnosed with diabetes mellitus, thyroid dysfunction or hyperprolactinemia; (6) control groups without healthy participants; (7) lack of exact data on the levels of serum INHB, AMH; and (8) studies with sample size < 10.

### Study selection and data extraction

**Fig. 1:**
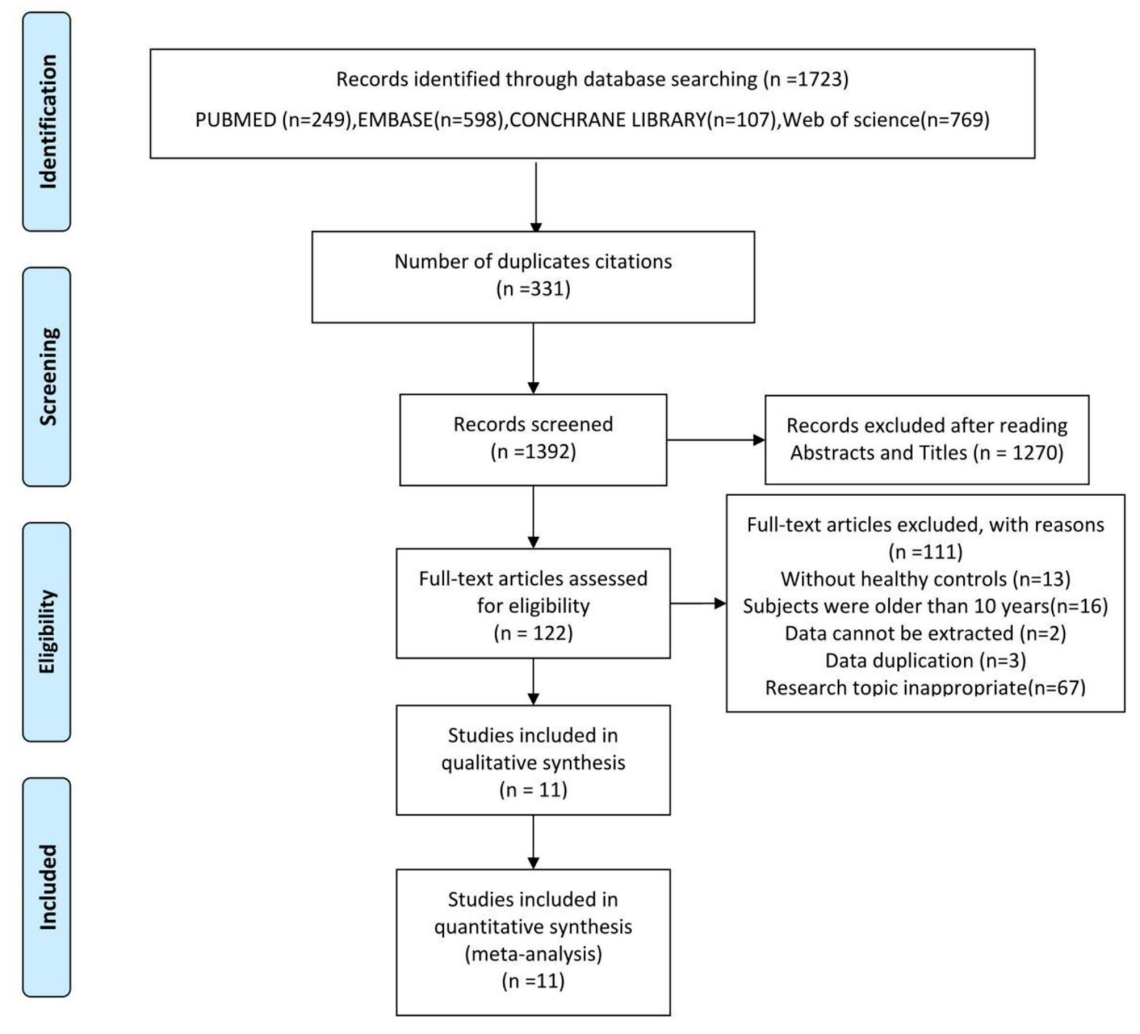
Flow chart of the selection process. *From: Moher D, Liberati A, Tetzlaff J, Altman DG, The PRISMA Group (2009). Preferred Reporting Items for Systematic Reviews and Meta-Analyses: The PRISMA Statement. PLoS Med 6(7): e1000097.*

Two researchers (MJ and YG) independently screened literature and extracted data. A third reviewer was consulted for disagreements between two reviewers (LH). The first step was to import references into EndNote, and to identify duplicates. Following that, abstracts and titles of published literature were screened and excluded based on inclusion criteria. As a final step, the full text of references was reviewed, and the studies were then excluded according to the inclusion and exclusion criteria. As seen in Fig. 1, a flowchart explaining the PRISMA selection process is presented. A complete set of data, including the levels of INHB and AMH [mean ± standard deviation (SD)], was extracted from the references, and all the data were double-checked by LH.

Each author was contacted and asked for the raw data when continuous variables were absent in reports. In instances where contact failed, standard deviation values were imputed from the median, quartileor ranges, if the data was only displayed graphically, the values were estimated using digital ruler software (Getdata Graph Digitizer, version 2.25) [19, 20].

### Quality Assessment

Based on the Newcastle-Ottawa Scale (NOS) star system, selected studies were assessed for quality [21], the foregoing studies were included with rating above 7.

### Assessment of risk of bias

Two researchers (MJ and YG) independently assessed the risk of bias using the Cochrane book. A third reviewer was consulted for disagreements between two reviewers (LH). In interpretingconclusions, these results were incorporated into the data analysis.

### Data synthesis

Meta-analysis was used to analyze the extracted data from the included studies. Due to the inconsistencies in existing references concerning units, the pooled standardized mean difference (SMD) and 95% confidence intervals (CI) were calculated to evaluate the associations between the levels of serum INHB, AMH and precocious puberty. Cochran’s Q two-sided test was used to test homogeneity in the studies [22]. In the case of I-square (*I*^*2*^) < 50%, a Mantel-Haenszel fixed-effect model was used. In the event of I^2^ ≥ 50%, a DerSimonian-Laird random-effects model was used [23, 24]. In an attempt to explain heterogeneity, we performed to identify associations between the levels of serum INHB, AMH and the characteristics of studies by conducting a subgroup analysis. Meanwhile, in an attempt to test the robustness of the pooled SMD, we performed sensitivity analysis by excluding individual study. The publication bias was measured on the method of funnel plot(in cases where the number of included references were ≥ 10) was used to test publication bias. All analyses were conducted by using RevMan software, and *P* < 0.05was considered to be statistically significant.

## Results

### Literature search

Among the 1392 articles initially reviewed, 1270 were excluded after reading titles and abstracts because they did not meet the inclusion criteria. 122 articles were included for full-text assessment, from which 111 were excluded: thirteen without healthy controls; sixteen involved subjects were older than 10 years; two involved data cannot be extracted; three repeated publication; seventy-seven research topic inappropriate. In total, 11 eligible papers (Fig. 1) were included in our analysis, all of which emphasized observational design and used individual data from 552 cases and 405 healthy controls [25–35]. The baseline characteristics, such as author, year, region, study design, sample size, chronologicalage (CA), bone age (BA), BA-CA of cases, duration of precocious puberty, drug usage, measuring method of AMH/INHB and primary conclusion included in the studies could be found in Table 1.

**Table 1:** Summary characteristics of studies and participants

No.	Study	Region	Study design	Sample size (n)		Chronological Age(y)		Bone Age		BA-CA of cases (mean, y)	Duration (y)		Tanner stage		Drug used	Measuring method of AMH/ INHB	Primary conclusion
				Cases	Control	Cases	Control	Cases	Control				Cases	Control			
1.	Crofton (2005) [32]	United Kingdom (Oxford)	cross-sectional study	24 (PT11, CPP13)	11	5.18±3.24	2.40±2.46	NA	NA	NA	NA		II	I	No	Doubleantibody ELISA	INHB increased in PT, remained unchanged in CPP
2.	Utriainen (2010) [33]	Finland (Kuopio)	cross-sectional study	PA 52	48	7.47±0.91	7.38±0.79	NA	NA	NA	NA		I	I	No	ELISA (Diagnostic Systems Laboratories Inc., Webster, Tex., USA)	AMH decreased in PA
3.	Sahin (2015) [34]	Turkey (Ankara)	cross-sectional study	65 (PT37, CPP28)	25	7.44±0.82	7.64±1.86	8.14±1.73	NA	0.7	PT	CPP	PT	CPP	No	ELISA (Immunotech, Beckman Coulter Inc., Brea, CA, USA)	AMH remained unchanged in CPP and PT
											2.11±2.71	5.34±5.89	II,III	II, III ,IV			
4.	Korkmaz (2016) [25]	Turkey (Izmir)	cross-sectional study	PP 28	20	7.62±0.77	7.42±0.88	NA	NA	NA	NA		II, III	I	NA	ELISA (Immunotech/Beckman Coulter instrument)	AMH increased in PP
5.	Nam (2017) [26]	Korea (Seoul)	cross-sectional study	CPP 98	55	8.4±0.5	9.4±0.5	9.9±0.6	9.8±0.4	1.5	NA		NA	NA	No	ELISA (Beckman Coulter Inc., Brea, CA, USA).	AMH remained unchanged in CPP
6.	Savas-Erdeve (2017) [27]	Turkey (Ankara)	cross-sectional study	45 (CPP21, PT24)	22	7.30±0.75	6.52±1.10	8.31±1.31	6.9±1.1	1.01	NA		NA	NA	No	ELISA (Anshlab AMH/MIS ELISA kit)	AMH increased in PT and CPP
7.	Grandone (2018) [35]	Italy (Naples and Trieste)	cross-sectional study	CPP 17	17	7±1.5	6.3±2.1	NA	NA	NA	NA		NA	I	No	ELISA (MyBioSource, San Diego, CA, USA)	AMH remained unchanged in CPP
8.	Brar (2018) [28]	US (New york)	cross-sectional study	PA 76	12	6.7±0.9	6.2±1.3	7.7±1.5	6.4±1.5	1.0	NA		NA	NA	NA	Gen II ELISA	AMH remained unchanged in PA
9.	Efthymiadou (2019) [29]	Greece (Patra)	cross-sectional study	PA 55	89	6.98±1.60	6.78±1.60	7.47±1.62	NA	0.49	NA		NA	NA	No	2-site ELISA (Diagnostic System Laboratories,Webster, Texas)	AMH increased in PA
10.	Jeong (2020) [30]	Korea (Cheonan)	cross-sectional study	CPP 48	35	8.43±0.46	8.01±0.54	10.48±0.6	7.79± 0.74	2.05	NA		II, III	I	No	Gen II ELISA (Immunotech, Beckman Coulter Inc., USA)	INHB increased in CPP, AMH remained unchanged in CPP
11.	Liu (2021) [31]	China (Beijing)	cross-sectional study	CPP 44	71	7.85±1.02	7.59±1.48	9.41±1.06	8.20±0.83	1.56	NA		II, III ,IV	II, III,IV	NA	Serum INHB was measured by a Gen II ELISA (Immunotech, Beckman Coulter Inc., CA, USA). Serum AMH was measured by an ELISA (Immunotech, Beckman Coulter Inc., CA, USA)	INHB increased in CPP, AMH remained unchanged in CPP

BA-CA, the difference between bone age and chronological age; INHB, Inhibin B;AMH, anti-müllerian hormone; CPP, central precocious puberty;PT, premature thelarche; PP, premature pubarche;PA, premature adrenarche; NA, not available; ELISA, enzyme linked immunosorbent assay.

### Results of Meta-analysis

#### Meta-analysis of INHB of precocious puberty (including CPP and PT)

**Fig. 2:**
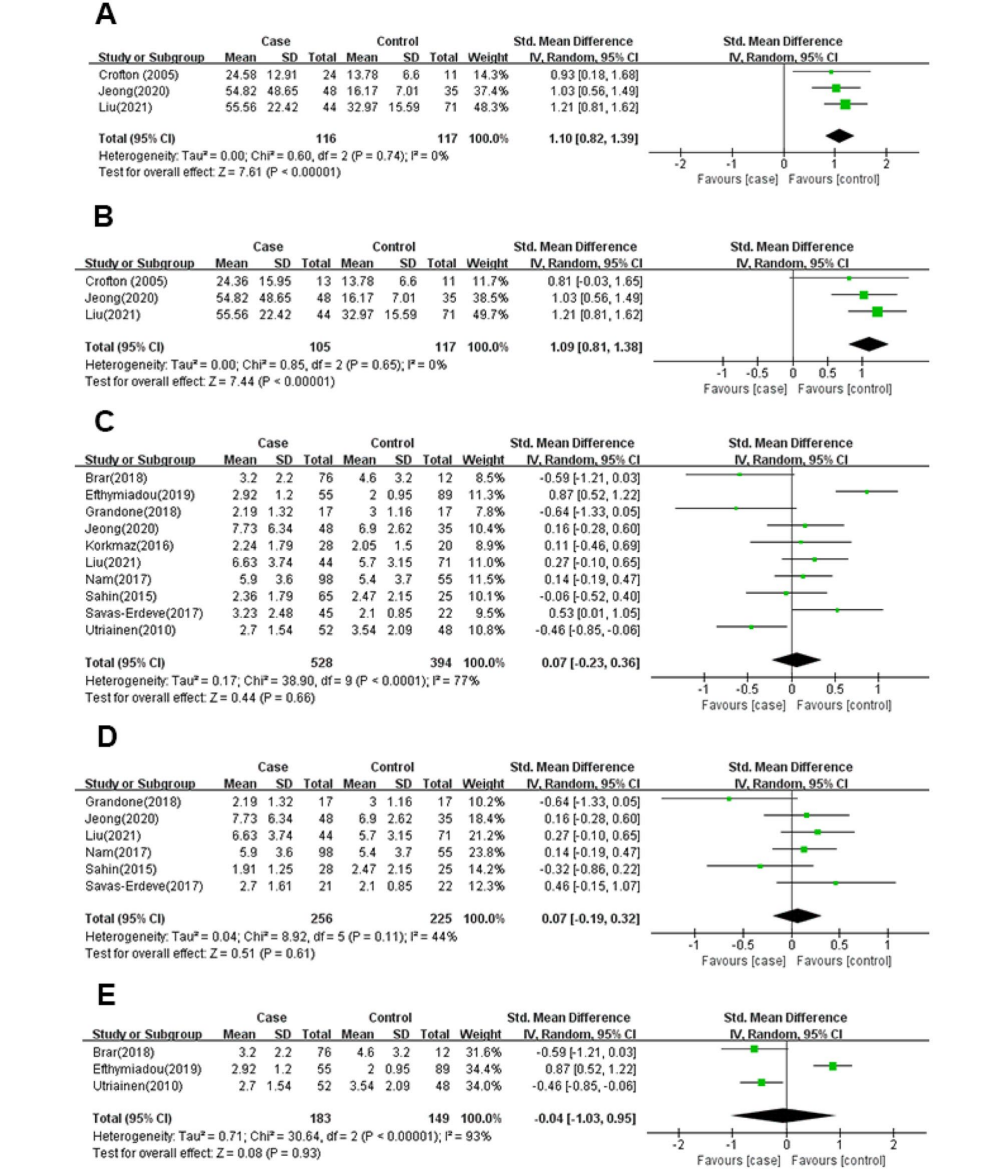
Forest plot of the levels of INHB or AMH in cases and healthy controls. Weights are from random effects analysis. (**A**) Meta-analysis of INHB of precocious puberty; (**B**) Meta-analysis of INHB of CPP; (**C**) Meta-analysis of AMH of precocious puberty; (**D**) Meta-analysis of AMH of CPP; (**E**) Meta-analysis of AMH of PA.CI, confidence interval; SD, standard difference.

Three studies (*n* = 233 participants) were included in the meta-analysis of serum INHB level of precocious puberty (Fig. 2A), and there was no heterogeneity among the studies (*I*^*2*^ = 0%; *P* = 0.74).INHB levels in serum were significantly increased in individuals who experienced precocious puberty (SMD:1.1; 95% CI: 0.82 to 1.39; *P* < 0.00001).

Three studies (*n* = 222 participants) compared the serum INHB level between girls with CPP and healthy controls (Fig. 2B), and there was no heterogeneity among the studies (*I*^*2*^ = 0%; *P* = 0.65). CPP was significantly associated with an increased level of serum INHB (SMD:1.09; 95% CI:0.81 to 1.38; *P* < 0.00001). Crofton’s study [36] disclosed that the level of serum INHB in girls with PT was higher than that in the control group (SMD:1.37; 95% CI: 0.42 to 2.31; *P* = 0.005).

#### Meta-analysis of AMH of precocious puberty (including CPP, PT, PP and PA)

Ten studies (*n* = 922 participants) were included in the meta-analysis of serum AMH level of precocious puberty (Fig. 2C), and there was significant heterogeneity among the studies (*I*^*2*^ = 77%; *P* < 0.0001).There was no significant association between precocious puberty and the level of serum AMH (SMD: -0.32; 95% CI: -0.23 to 0.36; *P =* 0.66).

Six studies (*n* = 481 participants) compared the level of serum AMH between girls with CPP and healthy controls (Fig. 2D), and there was low heterogeneity among the studies (*I*^*2*^ = 44%; *P* = 0.11).There was no significant association between CPP and the level of serum AMH (SMD: 0.07; 95% CI: -0.19 to 0.32; *P =* 0.61).Two studies (*n* = 108 participants) compared the level of serum AMH between girls with PT and healthy controls. Savas-Erdeve’s study [27] reported that the level of serum AMH in PT group was higher than that in the control group (SMD: 0.70; 95% CI: 0.10 to 1.30; *P* = 0.02). While Sahin’s study [34] showed that there was no significant association between PT and the level of serum AMH (SMD: 0.21; 95% CI: -0.30 to 0.72; *P =* 0.42). Korkmaz’s study [25] revealed that there was no significant association between PP and the level of serum AMH (SMD: 0.11; 95% CI: -0.46 to 0.69; *P =* 0.70).

Three studies (*n* = 332 participants) were included in the meta-analysis of the level of serum AMH of PA (Fig. 2E), and there was significant heterogeneity among the studies (*I*^*2*^ = 93%; *P* < 0.00001).There was no significant association between PA and the level of serum AMH (SMD: -0.04; 95% CI: -1.03 to 0.95; *P =* 0.93).

### Results of Subgroup Analysis

There was significant heterogeneity among the studies including in the meta-analysis of serum AMH level of precocious puberty. To search for the sources of heterogeneity and more accurately assess the differences between girls with precocious puberty and healthy controls, subgroup analyses were conducted by geographical location, number of cases, chronological age of cases, the difference between bone age and chronological age of cases, detection reagent (Table 2).

**Table 2:** Subgroup analysis to investigate the relationship between geographical location, number of cases, chronological age of cases, the difference between bone age and chronological age of cases, detection reagent and serum AMH

Subgroups	No. of studies	*I* ^*2*^	SMD (95%CI)	*P*
**Geographical location**				
Europe	3	93%	-0.05 (-1.07, 0.96)	0.92
North America	1		-0.59 (-1.21, 0.03)	0.06
Central Asia	3	30%	0.19 (-0.17, 0.54)	0.31
Asia	3	0%	0.19 (-0.03, 0.40)	0.09
**Cases**				
< 50	5	46%	0.15 (-0.16,0.45)	0.35
≥ 50	5	87%	0.00 (-0.51,0.52)	0.99
**Chronological age**				
< 8	8	82%	0.03 (-0.36, 0.43)	0.87
≥ 8	2	0%	0.15 (-0.12, 0.41)	0.28
**BA-CA**				
< 1.5	4	86%	0.22 (-0.41, 0.84)	0.50
≥ 1.5	3	0%	0.19 (-0.03, 0.40)	0.09
NA	3	41%	-0.33 (-0.73, 0.08)	0.12
**Detection reagent**				
Beckman	6	19%	0.07 (-0.13, 0.27)	0.50
Others	4	90%	0.10 (-0.65, 0.85)	0.80

BA-CA, the difference between bone age and chronological age; SMD, standard mean difference; NA, not available.

As for geographical location, there was no significant association between precocious puberty and the level of serum AMH in Europe (*P* = 0.92), North America (*P* = 0.06), Central Asia (*P* = 0.31), Asia (*P* = 0.09) subgroup. The heterogeneity of Central Asia subgroup decreased (*I*^*2*^ = 30%), and there was no heterogeneity in Asia subgroup (*I*^*2*^ = 0%).

As for number of cases, serum AMH level was not associated with precocious puberty in the subgroup with cases < 50 (*P* = 0.35) and the subgroup with cases ≥ 50 (*P* = 0.99). The heterogeneity was reduced in the subgroup with the number of cases < 50 (*I*^*2*^ = 46%).

As for the mean chronological age of cases, there was no significant association between precocious puberty and the level of serum AMH in the subgroup with the mean age of cases < 8 (*P* = 0.87) and the subgroup with the mean age of cases ≥ 8 (*P* = 0.28), and no heterogeneity was found in the subgroup with the mean age of cases ≥ 8 (*I*^*2*^ = 0%).

As for the difference between bone age and chronological age of cases, in the subgroup with the difference < 1.5, there was no correlation of AMH level in girls with precocious puberty and healthy controls (*P* = 0.50), and the same conclusion was found in the subgroup with the difference ≥ 1.5 (*P* = 0.09) and the subgroup with the difference not available (*P* = 0.12). There was no heterogeneity in the subgroup with the difference ≥ 1.5 (*I*^*2*^ = 0%), and the heterogeneity was reduced in the subgroup with the difference not available (*I*^*2*^ = 41%).

As for detection reagent, in the subgroup with detection reagent of Beckman Coulter (*P* = 0.50) and the subgroup with detection reagent of others (*P* = 0.80), the AMH level was not associated with precocious puberty. The heterogeneity of the subgroup with detection reagent of Beckman Coulter decreased (*I*^*2*^ = 19%).

### Sensitivity analysis

The studies were removed one by one to check the stability and reliability of the meta-analysis results. As for INHB and AMH levels, there was no qualitative change in the total effect size after removing the studies one by one, indicating that the meta-analysis results were stable and reliable.

### Risk of bias of included studies

**Fig. 3:**
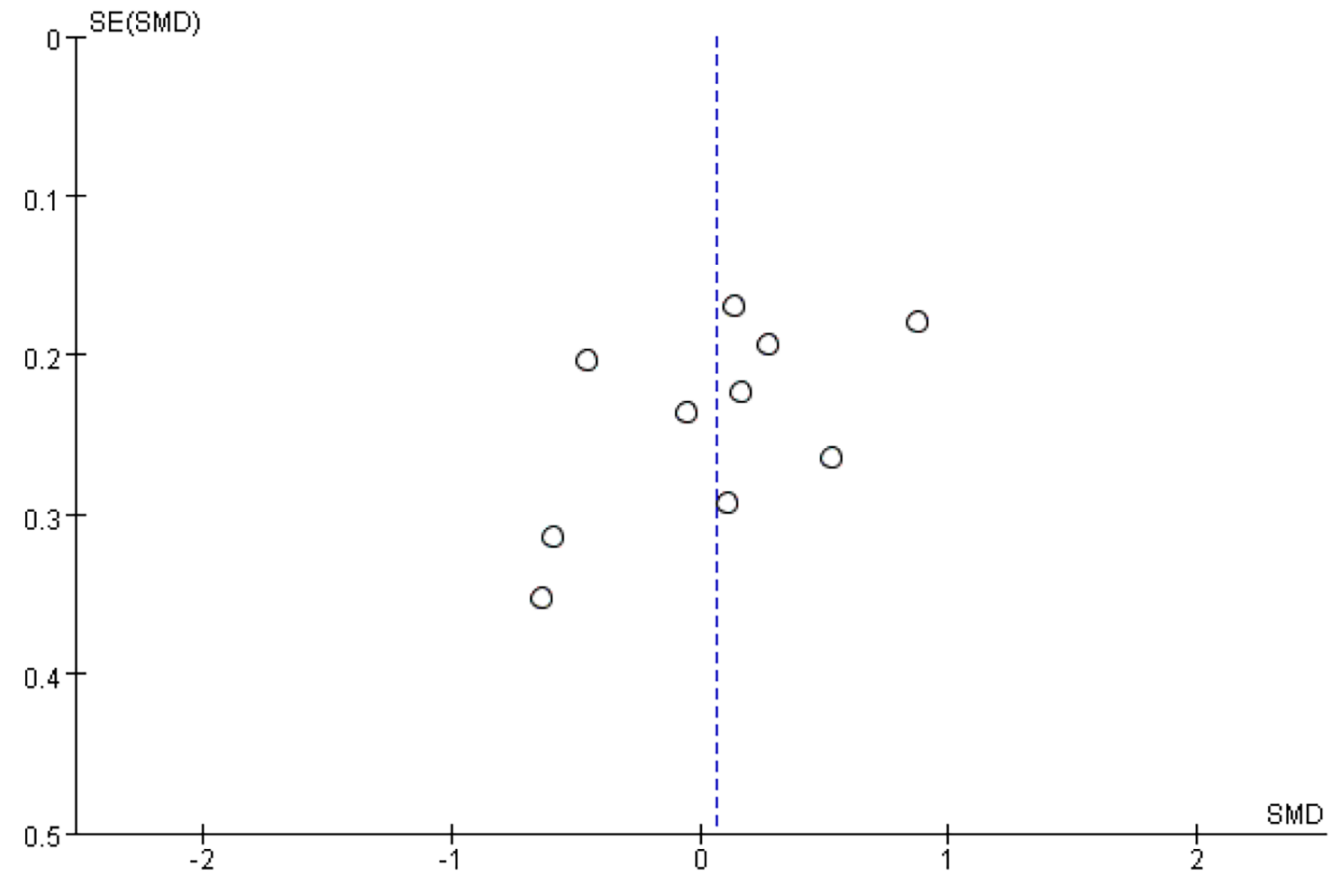
Funnel plot analysis for AMH to detect publication bias

The funnel plot method was used to detect publication bias,regarding AMH, the shape did not being clearly asymmetric (Fig. 3), indicating publication bias of this meta-analysis was not evident. As the Cochrane Handbook for Systematic Reviews of Interventions (www.cochranehandbook.org) stated that multiple studies not gathered with 10 studies yield unreliable results, publication bias was not assessed for INHB.

### Synthesis of results

A total of 11 studies featuring 552 girls with precocious puberty and 405 healthy girls were selected for analysis. The meta-analysis showed that the INHB level of precocious puberty [including central precocious puberty (CPP) and premature thelarche (PT)] were significantly increased. While there was no significant association between precocious puberty [including CPP, PT, premature pubarche (PP) and premature adrenarche (PA)] and the level of serum AMH.

## Discussion

Until now, there has been no meta-analysis that has reported the relationship between level of serum INHB, AMH and precocious puberty. This meta-analysis selected 11 studies and covered 957 girls. The subgroup analyses based on geographical location, number of cases, chronological age of cases, the difference between bone age and chronological age of cases, detection reagent were performed. This meta-analysis indicated that the level of serum INHB in girls with precocious puberty (including CPP and PT) was significantly higher than that in healthy controls, however, no significant difference was observed regarding the level of serum AMH between girls with precocious puberty (including CPP, PT, PP and PA) and healthy controls.

The early diagnosis and treatment of precocious puberty are particularly crucial. Changes of hormone levels, acceleration of physical growth in advance, and epiphyseal fusion ahead of time lead to the final adult height in girls with precocious puberty be lower than that in healthy girls. Furthermore, the secondary sexual characteristics of precocious children appear in advance, but the mental level and psychological development do not, that is, premature sexual characteristics and genital development will lead to psychological and social disorders in immature children [37]. The “8th Korea Youth Risk Behavior” online survey found that early sexual behaviour and teenage pregnancies highly correlated with premature menarche [38]. Despite being considered a benign condition, current studies suggested that PA might be associated with insulin resistance, a higher risk of heart disease, ovarian hyperandrogenism and a higher likelihood of developing polycystic ovarian syndrome (PCOS) [39–41].

INHB is useful for determining precocious puberty. As puberty begins, serum INHB level rises dramatically [14], a rising INHB level indicates that more follicles are being recruited as puberty progresses because INHB is only produced by small antral follicles in the ovary [42]. In this study, girls with precocious puberty, including those with CPP and PT, had higher INHB level than healthy controls. Andmany previous studies have suggested INHB as a potential biomarker of precocious puberty. According to study reported by Shested et al., INHB does not outperform estradiol and luteinizing hormone (LH) in terms of predicting pubertal progress, but it may indicate the degree of follicular maturation and may be used in conjunction with ovarian ultrasonography to assess puberty [42]. De Fililppo et al. found that combining basal LH levels with basal INHB values offers a trustworthy way to spot a developing kind of CPP in girls [43]. These findings imply that INHB reflects pubertal state and rises as puberty progresses, therefore, INHB might be related to the onset of precocious puberty and might be used as the auxiliary screening tool for early detection and treatment of precocious puberty (especially CPP and PT). However, the specific differential diagnosis of CPP and PT requires further examination.

However, the AMH level was not associated with precocious puberty in girls. The level of serum AMH in females is lower than in males during postnatal life, but it starts to rise from 6-8 years old, peaks in late adolescence, and then starts to decline along their reproductive life [44]. In actuality, AMH decreases with puberty, perhaps due to ovarian maturation and the development of antral follicles from small antral follicles [16, 45]. In women who are in reproductive age, there is a close association between AMH serum level and small antral follicular reserve [46]. Furthermore, AMH serum level is higher in women and adolescents with PCOS compared with healthy controls [47, 48], and indicates how severe the condition is. Additionally, studies have suggested that the detection of AMH serum level could be used as a PCOS diagnostic tool [49, 50]. The level of AMH prior to pubertal commencement, however, does not correlate with the age at which puberty begins [16]. In this study, there was no discernible difference in AMH level between girls with precocious puberty (including CPP, PT, PP and PA) and healthy controls. All the above indicated the AMH level was connected with the onset of puberty but not with the age of onset.

Subgroup analysis was conducted to further explore the source of heterogeneity and to assess the correlation between a high level of AMH and precocious puberty in a more accurate manner. Among the cases in this meta-analysis of the correlation between AMH level and precocious puberty, heterogeneity may be present due to region, number of cases, chronological age of cases, the difference between bone age and chronological age of cases, as well as detection reagent use. (1) It is likely that a person’s diet, lifestyle, and economic level will affect how their ovary matures differently in different regions; (2) This meta-analysis included only case-control studies. Co-founders can have a negative effect on the results of cases-control studies if the number of cases is enormous; (3) Physiological age plays a important role in ovarian reserve function, thus chronological age was considered as one of the sources of heterogeneity; (4) The difference between bone age and chronological age of cases can reflect the severity and course of the disease, and AMH level was measured at different stages in different studies. Thus the difference between bone age and chronological age of cases might be one of the sources of heterogeneity; (5) The Gen II ELISA of AMH developed by Beckman Coulter was more sensitive than the older version [51]. Therefore, the detection reagent use was regarded as another source of heterogeneity. Moreover, sensitivity analysis was conducted in this meta-analysis, as for INHB and AMH levels, there was no qualitative change in the total effect size did not qualitatively change after removing the studies one by one, suggesting that the meta-analysis results remained stable and reliable.

Nonetheless, there are a number of limitations to this meta-analysis should be acknowledged. First of all, obvious heterogeneity existed among the AMH level of original studies due to differences in sample size, background of subjects, and the detection reagent used to detect the level of AMH. Secondly, there was no association between precocious puberty and the level of serum AMH, possibly due to the different usage of detection reagents. Furthermore, INHB kits differ widely between lots, potentially also causing bias to the results. In addition, insufficient number of references was also one of the reasons. Therefore, more references are needed to verify this result. Additionally, it is unclear whether the main confounding factors (e.g., dietary habit and physical activity) affecting the ovarian reserve function of girls in original studies were adjusted for. Finally, the references included in this study were all case-control studies, which would limit causal inference. And it takes many years for a child to reach puberty, therefore clinical evaluation and follow-up are still essential. To confirm the clinical importance of AMH and INHB in precocious puberty, and to ascertain the long-term reproductive function in girls with precocious puberty, more studies (especially cohort studies) are required. In these circumstances, we therefore recommend a conservative interpretation of our conclusions.

## Conclusion

Overall, this systematic review and meta-analysis showed that the INHB level, but not the AMH level, was significantly higher in subjects with precocious puberty than healthy controls. These meant that the level of INHB was associated with a number of clinical indicators of precocious puberty and could be a reliable marker for girls with disease at an early stage. While the AMH level somewhat influenced the hypothalamo-pituitary-gonadal axis, but more research was needed to investigate its clinical implications in precocious puberty. Furthermore, we recommend that the clinical indicators like FSH, LH, and INHB should be evaluated and comprehensively investigated to ensure that early diagnosis and medical intervention, and to minimize the risk of physical, psychological and social disorders in immature girls with precocious puberty.

## Data Availability

All data is available from the corresponding author on reasonable request.

## Abbreviations

AMH: anti-müllerian hormone
BA: bone age
CA: chronological age
CI: confidential interval
CPP: central precocious puberty
ELISA: enzyme-linked immunosorbent assay
FSH: follicle-stimulating hormone
GnRH: gonadotropin-releasing hormone
HPGA: hypothalamic-pituitary-gonadal axis
INHB: inhibin B
LH: luteinizing hormone
MOOSE: Meta-analysis of Observational Studies in Epidemiology
NOS: Newcastle-Ottawa Scale
PA: premature adrenarche
PCOS: polycystic ovarian syndrome
PP: premature pubarche
PRISMA: Preferred Reporting Items for Systematic Evaluation and Meta-Analysis
PT: premature thelarche
SD: standard deviation
SMD: standardized mean difference
